# Essential Nature of Caries

**Published:** 1867-10

**Authors:** H. R. Noel

**Affiliations:** Professor of Physiology, Baltimore College of Dental Surgery.


### AMERICAN JOURNAL

OF

### DENTAL SCIENCE.

Vol. I. THIRD SERIES—OCTOBER, 1867. No. 6.

### ORIGINAL COMMUNICATIONS.

### ARTICLE I.

*Essential Nature of Caries.*

(Continued.)

By H. R. Noel, M. D., Professor of Physiology,
Baltimore College of Dental Surgery.

In the case of dentine, the calcification begins with the "*cells or bodies*" that are to form the intertubular substance, so-called, and gradually closes in around and forms what are known in the dry tooth, as the walls of the dentinal tubes. But a portion remains uncalcified, and the uncalcified portions are the fibrils or fibrillæ and pass back to be connected with the proper structure of the pulp. Now what are those fibrils or fibrillæ? What are those soft, fleshy bodies or filaments that fill up, what were formerly called the dentinal tubes? They are but modified "elongated cells or bodies," yet uncalcified, but as the tooth advances in age, the calcification slowly progresses and the size of each fibrilla becomes less—while the walls are thickened. What is lost in fibrillæ, is gained in calcified dentine, or walls; and the dentine becomes *denser*.

The dimunition in the size of the fibrillæ, and the diminution in the size of the pulp, are alike occasioned by the direct calcification of the elongated cells or bodies. These cells therefore exist, always between the pulp and dentine formed, and between fibrils and dentine surrounding ; and these fibrils themselves are nothing more than these "elongated cells or bodies" extending from periphery of the dentine to the pulp proper and into the pulp, and remaining uncalcified ; but liable at any time to be calcified, more or less, and thus lose in size, but gain in dentine surrounding. If, while still uncalcified they are fibrillæ, so equally true is it that once calcified, they form true dentine.

Tomes and Beale support this view. Now we wish to be clearly understood here, and shall repeat in substance what we have just asserted, as upon a clear understanding of this portion of our article, hinge the deductions we intend making, as regards the essential nature of caries.

The relation therefore between fibrils, and what are called the *walls of the dentinal tubes*, but which we shall call *the last formed dentine*, is simply one of organic continuity ; the fibrils in the recent or fresh tooth occupy and completely *fill* the tubes, and the walls of these so-called tubes are but calcified portions of the fibrils ; or in other words, the openings and spaces, called *Dentinal Tubes* in dry teeth have really no actual existence in the fresh tooth, but are occupied by a tissue identical in every respect with that which has been calcified to form the dentine ; and the said tissue, though remaining uncalcified, is liable at any time to calcification, and the fibrils may be obliterated or only have their size diminished : in either case dentine is the product resulting. Now if we begin at the pulp and go out to the periphery, we would say that these fibrils branch, divide, anastomose and grow smaller as they approach the periphery ; but this is not true physiologically ; and we should rather say that the fibrillæ begin by very minute filaments at the periphery, and uniting as they ran towards the pulp, terminate by larger ends in the pulp proper.

Mr. Tomes carefully prepared specimens ; and to satisfy himself upon this point, Dr. Beale did also. The former carefully removed the pulp from a fresh tooth, and found the fibrils broken, torn, &c., showing by their projecting lengths that they had been withdrawn from the dentine. He fractured a fresh tooth, and examined a thin slice of it: here also the microscope showed the torn ends of fibrils projecting from, and extending into the fracture, between the two fragments of dentine; some passed directly across the fracture from one fragment to the other. These fibrils sometimes, though very rarely, extend into the enamel.

The enamel rods or prisms may be compared to cells or fibres, where cellwall or sheath and contents of cell or body of fibre are alike calcified. They are solid, dense, compact bodies, and if they possess any tubes at all, belonging intrinsically to them, the highest powers of the microscope have signally failed to demonstrate the fact.

We would say then that the vital endowments of enamel are nothing, the vital powers of enamel nothing; that enamel has absolutely *no vitality.* When once calcified, we emphatically deny that enamel can possibly be amenable to any laws, regulating the actions performed in vitalized structures; it changes in obedience to physical violence, or chemical action alone; and once fully formed, is forever beyond the dominion of the vital forces. Enamel may be fractured, may be worn away,—may be dissolved by acids, may be stained or discolored by fluids, and the same is equally true of the marble slab of a table.

The "elongated cells" "rods" or "bodies" were once vital, and contained active nuclei and nucleoli, as centres of vitality, points of departure for vital forces, but these have become calcified, have died, and having lost their vitality are no more capable of exhibiting vital phenomena, than grains of sand. The enamel itself, as enamel, is simply a mineral, an inorganic body, so far as its relations to vitality are concerned.

A careful study of this subject has led us to the conclusion, that enamel is not a vitalized structure in the true sense of that word, and therefore *absolutely incapable of vital phenomena.* But *caries* often occurs in *enamel primarily.* We wish this point carefully remembered.

Now as regards dentine, the fibrils redeem it from the class in which we place enamel, for the fibrils have nuclei and the nuclei are centres of vital power, of formations and transformations; and of chemico-vital changes, &c., moreover nutritious fluids do pass by osmosis along these fibres, even to their ultimate branchings and ramifications on the periphery of the dentine. Sensation is conveyed by these same fibrils, even from the very periphery of the dentine to the nerves of the pulp. This will however be more fully developed in its proper place, and we will endeavor to assign to dentine its full share of vitality, and then measure the influence of this vitality in the process we term caries.

In the present state of physiological knowledge are we justified in stating that any structures, tissues, &c., are truly vitalized unless they possess live, active working nuclei? If nuclei are the germs, are the centres of vital power, the centres of the forming and transforming forces; the centres of the powers of assimilation, growth, developement, &c., the *vitality* of a tissue would be in direct ratio, to the number and activity of its nuclei.

What are the relative numbers and what the activity of the nuclei in the dentinal fibrillæ or fibrils? What are the vital phenomena they exhibit and what are the changes they undergo? Eliminating all of the physical, all of the purely chemical phenomena, a close, rigid, logical reasoning forces us to the wall here also, and we must decide that dentine is vitalized in proportion to the nuclei of its fibrils and that the calcified cells and calcified nuclei have passed from the domain of the vital forces.

Fibrillæ are vitalized, but the calcified portions are non-vitalized.

That the calcified dentine is not a truly vitalized structure, seems to be beyond question, for its "Germinal Centres,"—"Germinal Material," nuclei and nucleoli are calcified and therefore utterly incapable of exhibiting vital phenomena.

The fibrils contain nuclei, &c., are vitalized, and do exhibit vital phenomena, and to a limited extent obey the laws of vitalized structures; but once calcified, they are forever beyond the domain of the vital, and any change therefore taking place in dentine proper results from far other than vital forces, inherent in the dentine. We make this distinction therefore, that changes in fibrils may result from vital or chemico-vital forces,—but changes occurring in dentine proper, result from a force extraneous, external, and not from any inherent power; unless—and here is a nice point,—unless the changes begin on the inner sides of the *so-called walls* of the dentinal tubes; i. e. begin at that vaguely defined boundary, between the calcified walls and uncalcified but still calcifying fibril.

If caries always begins here, at this point, or nebulous line between dentine and fibril, it is in close proximity to nuclei and we would infer that it was more or less modified by or dependent upon the vital force contained in the nuclei of the fibrils.

Does caries begin there? We will let Mr. Tomes answer the question.

"The connecting material ("intertubular substance") is usually first and the *walls* of the tubes *last*, to become disintegrated."—Dental Surg., p. 359.

Before passing on to a more critical examination of the subject, we propose to give the views of Dr. Beale as to the vital changes going on in dentine and enamel.

"These dental tissues, of all textures in the body, are those which undergo the least amount of change, and the statement that the material of which our teeth are composed is being continually removed and renewed, is a mere assertion, and utterly unsupported by facts or by sound arguments."—Structure, Growth and Life, p. 149.

"There is no evidence of the addition and removal of material going on in the enamel and dentine after the completion of their formation, and it is probable that the matter upon which the hardness of these tissues depends is not removed at all after its deposition."—*Idem p.* 150.

Neither then the organic base can be.

"When the enamel thus produced has reached a certain thickness, the germinal matter-nucleus, dies, the tooth passes through the gum, and the vital changes occurring in the enamel *cease forever.*"—*Idem p.* 175.

"Nor is there any room whatever for believing that the fully formed enamel is nourished or appropriates nutrient matter."—*Idem p.* 175.

Our readers will please mark well these statements, as they have a direct bearing upon the vital theory of caries.

"Now it would be said that when the vessels of the pulp are destroyed and nutrient matter no longer permeates the tooth structure, the entire tooth is dead; but it is doubtful if in such a case the enamel, the outer part of the dentine, the inner part of the cementum, are more *inanimate* than when the *tooth* formed an integral part of the living body, and was supplied with nutriment from the same living blood as the soft parts."—*Idem p.* 194

This is but a logical sequence of Dr. Beale's theory, of Structure, Growth, &c., and we here accept his views as applied to the teeth, for upon this point, practical experience and collateral evidence sustain his physiological researches!

The calcification of the "Enamel Columns," "rods," "elongated nucleated bodies," whatever we may call them the calcification of their nuclei and germinal centres, removes them from the domain of vital to that of nonvitalized structures.

The calcification of the "elongated cell," "rods," &c., of the superficies of the dental pulp, to form dentine, removes these also from vital to nonvitalized structures, so far as the calcified part is concerned. Where then exists the vitality of dentine? We answer in the fibrils alone. The fibrils exhibit all the necessary phenomena of vitality; nutrition, assimilation, conduction of sensation, &c.

But where does caries usually begin?

As regards cementum, we have little or nothing to say, as it is rarely if ever carious, unless the gum be removed, and external agents brought in contact with it; or caries extends from dentine to cementum. The consolidation of decay—is, even in cementum, external and therefore not a vital agency. This we take wholesale from Mr. Tomes.

"The disease may extend to, or may even commence in the cementum in teeth, from the *neck of which* the *projecting gum* has *been* removed; but these *are exceptional cases.—Dental Surgery, p.* 353.

This we implicitly believe, and only remark that the *agent* producing decay is an external one necessarily, as decay never occurs in cementum *first, unless* the gum be removed and the neck of the tooth thus denuded.

As regards structure, cementum is allied to bone; but Dr. Beale thinks dentine and enamel are homologous with epithelial structures.

"I look upon both enamel and dentine as calcified epithelial structures."—*Structure, Growth, and Life, p.* 173.

But the practical bearing of these refinements, as regards homology, are too slight to detain us in any discussion of fanciful theories.

We have omitted many points of interest in the history of the developement of the teeth; and also much of importance as regards defects of calcification, yet we have been too long in coming to the main point or question, i. e. caries. The fact is deprecated, but the work was absolutely required, before we could clearly comprehend the full bearing of the question, "the Essential Nature of Caries."

### Predisposing Causes.

We give these as Mr. Tomes gives them; and as they have some relation to the question, it is well to repeat them.

*Enamel Defective.*—(1.) In quantity. (2.) In quality. Quantity deficient, as (*a*) pits, indentations, transverse groves, &c.—(*b*) defect in amount in the cusps, cusps small and enamel thin over the whole crown; (*c*) deep fissures between the cusps, extending down towards the dentine.

Quality.—Defective calcification; (*a*) defective fusion of the sheaths of the enamel rods, (very doubtful,) (*b*) defective calcification of the central portion of the rods; (*c*) small linear cavities through the central axis of the rods, (very rare.)

(3.) Blending of defects in both quantity and quality.

*Dentine Defective*—(*a*) granular and imperfectly calcified condition of the tissue immediately on the line of union of enamel and dentine; (*b*) Globular dentine, and interglobular, cellular &c., cells not calcified or badly calcified; (*c*) cavities in the dentine; (*d*) dentinal tubes (fibrillæ) running into cavities and dilating (bad at best, this statement, and not sustained by the anatomy of the part,) (*e*) "diminished density, and increased porosity," i. e. imperfect calcification. Well calcified, dense, hard teeth, resist decay; badly calcified teeth, soft and porous in structure, are more liable to decay, and decay more rapidly after once the process begins. Defects of this kind in enamel, are directly predisposing causes of decay,—defects in dentine however are not, and only give an easier substance *for already existing caries to act upon*, but never primarily predispose to decay, *unless* the dentine be uncovered by enamel, and the agent or cause has direct access to the dentine.

If the enamel be perfect—if it should accurately cover the dentine—if it be of normal thickness, we are decidedly of the opinion that caries of the dentine will not occur, *no matter* what the imperfections of the dentine may be. In plainer language—caries is produced by an external agent, and therefore if dentine be thoroughly protected by enamel, *decay never occurs there*, i. e. in dentine primarily.

A singular statement and admission is made by Mr. Tomes, as follows.

"Although the presence of defects in the structure of the dentine, no doubt contributes to hasten the destruction of teeth when attacked by caries, yet as a predisposing cause, *they are secondary in importance* to similar *faults* in the organization of the *enamel*."—*Dental Surg., p.* 348.

Now this is the result of Mr. Tomes's practical experience and in direct opposition, as we shall find, to his theory of decay. By experience he has learned whence to expect the enemy; evidently an external agent, a force not vital as it is exterior to the tooth. A vital force would attack dentine first, but here the approach through enamel is dreaded.

We endorse heartily, Mr. Tomes' apprehension of danger from an external agent.

We have thus far, arrived at three very essential conclusions:

1. The enamel is not vitalized.
2. The dentine (calcified) is not vitalized.
3. The agent causing decay is an external one.

We will now make a searching examination of the fibrils and a rigid analysis of the parts they play in caries. Let us be clear and explicit. We have shown that they are organically continuous with the proper tissue of the pulp; that they are nuclear fibres, or fibrillæ; they are the "elongated cells," "rods" and "bodies" of Tomes, Kölliker, and Beale, &c., which though not yet calcified are liable to be at any time. We also asserted that they were organically continuous with the calcified portion *called Dentinal Tubes*, and that a thin stratum, of, as it were, neutral ground, existed between wall and fibril; a nebulous line, possibly neither dentine proper nor fibril proper, but marking the transitional stage between the two; between the calcified and uncalcified, i. e. *a changing calcifying boundary.*

That these fibrillæ or fibrils are modified "elongated cells" or "bodies," cannot be doubted; they have acquired some new characters, but lost none of their essential characteristics, as elongated cells; their essential nature is the same. They contain nuclei, nucleoli, &c., therefore contain the indispensable conditions of vital action. What are the phenomena they exhibit?

1st. Passage of a nutrient fluid along the fibrils, and this in obedience to the force developed, in the chemico-vital processes of nutrition and assimilation, and in no manner dependent upon the heart; the force drawing plasma from the vessels of the pulp, is the same force that produces circulation in a vegetable cell, dependent absolutely upon the vitality of the *uncalcified working* nuclei; should these nuclei die, should they be calcified, any passage of fluid to or around them is impossible, except by a physical force, as imbibition, or capillary attraction.

The vital element is lost.

2nd. Solidifying of the fibrils—calcification of the fibrils.

Tomes, Beale, Arthur, &c., assert, and dogmatically assert this change in the fibrils. Tomes calls it, the "zone of consolidation;" Arthur calls it, the "consolidation of dentinal tubes and fibrils in the space immediately beneath the caries." Both designate it as an effort of nature to arrest caries; they grant the complete obliteration of the tubes or rather fibrils; that is, they assert that a conservative effort is made to arrest caries; that this effort consists in the consolidation (calcification) of the dentinal tubes (fibrils,) to such an extent, that the dentine is denser, harder and more impervious to attacks, than even before the consolidation took place. Of course the degree, or the completeness of the consolidation, is the measure of its power of resistance. We call attention, marked attention to a quotation from Mr. Tomes' work.

"The zone of consolidation *cuts off* and *isolates* the diseased from the healthy portion of the tooth, and its production must be regarded as an attempt on the part of nature to circumscribe and limit the mischief."—*Dental Surg.*, *p.* 364.

(To be Continued.)

